# Editorial: Molecular Targeted Therapy in Oncology: Lessons From Pharmacogenetics and Pharmacoepigenetics

**DOI:** 10.3389/fmolb.2022.822188

**Published:** 2022-03-15

**Authors:** Saber Imani, Matteo Becatti, Md. Asaduzzaman Khan

**Affiliations:** ^1^ China Regional Research Center, International Centre for Genetic Engineering and Biotechnology Taizhou, Jiangsu, China; ^2^ Department of Oncology, Affiliated Hospital of Southwest Medical University Luzhou, Luzhou, China; ^3^ Department of Experimental and Clinical Biomedical Sciences “Mario Serio”, University of Firenze, Firenze, Italy; ^4^ The Research Center for Preclinical Medicine, Southwest Medical University, Luzhou, China

**Keywords:** cancer, molecular targeted therapy, pharmacogenetics, pharmacoepigenetics, signaling pathways, drug resistance

The Human Genome Project is moving away from basingclinical decisions on signs and symptoms of diseases to a new concept of medicine. Pharmacogenetics and pharmacoepigenetics are multidisciplinary research areas of “personalized genomic medicine”, representing a new approach to healthcare aimed at customizing patients’ medical treatment according to their individual genomic profile.

In the current Research Topic (RT), we provide an overview on the new trends, achievements, and challenges lying ahead in cancer. Original articles and reviews summarize the current knowledge on genomics and epigenetics modifications to study therapeutic approaches, biomarkers, and mechanisms involved in diseases and drug resistance.

In recent years, the multi-OMICS data have provided a platform linking the cancer-specific genomic/epigenomic alterations to interconnected transcriptome, proteome, and metabolome networks which underlie the molecular-targeted therapies in oncology (Jung et al., 2021). The development of high-throughput sequencing technologies and artificial intelligence have allowed a large data explosion and systematic study of the cancer genome. For the first time, data were available for complete genome sequences in a large number of cancer types. Open data have promoted the advance of scientific discovery. The era of genomics and big data has brought the need for collaboration and data sharing in order to make effective use of this new knowledge. Consequently, the storage and analysis of these data in an efficient manner pose a major challenge to researchers. In this RT, five studies that employed publicly accessible databases useful for cancer diagnosis and prognosis are presented (Deng et al.; Fan et al.; Feng et al.; Xiang et al.; Zhang et al.). This approach provides a new opportunity to optimize treatments by understanding the basis of biological mechanisms and utilizing genomic/epigenomic contributions to treatment response. In the context of tumor-specific therapeutical biomarkers in immunotherapy, programmed death-1 homolog (PD-1H) is a new regulator of T cell-mediated immune responses (Villarroel-Espindola et al., 2018). Chen et al. showed that PD-1H could be a prognostic indicator and a potential immunotherapeutic target in human esophageal squamous cell carcinoma (ESCC) patients (Chen et al.).

Tyrosine kinases receptors (TKRs) are transmembrane proteins acting as signal transducers that regulate essential cellular processes such as proliferation, differentiation, metabolism, and cell death. Alterations in the TKR pathway occur in a broad spectrum of cancers, underlying their key role in cancer progression and as therapeutic targets (Krause and Van Etten, 2005; SudheshDev et al., 2021). In this RT, Wu et al. investigated the neurotrophic tyrosine receptor kinase (NTRK) gene fusion status in triple negative breast cancer (TNBC) patients (Wu et al.). In the same line, Yang et al. investigated the function of ELK1 transcription factor in pancreatic cancer progression (Yan et al.), demonstrating the key role of ELK1 in cancer cell proliferation and invasion.

Notch and Wnt signaling are other critical pathways involved in cell fate decision, cell proliferation/differentiation, and cancer progression. In addition, two review papers summarized the current knowledge on the molecular mechanisms by which Notch3 and Wnt modulate cancer stemness and chemoresistance, metastasis, and angiogenesis. Xiu et al. suggested potential treatment strategies to block Notch3 signaling, such as non-coding RNAs, antibodies, and antibody-drug conjugates (Xiu et al.). Furthermore, the article by Xie et al. summarized the current knowledge and recent advances on the Wnt signaling pathway in head and neck squamous cell cancer (Xie et al.), suggesting that Wnt could be a new potential target for innovative therapeutic approaches.

Oxaliplatin is a popular therapeutic agent against cancer associated with neuropathic pain. The genetic and epigenetic mechanism of oxaliplatin on neuropathic pain has been discussed by (Branca et al.). Understanding the ability of oxaliplatin to alter the genetic and epigenetic profiles of neural cells might be helpful to find new treatments for neuropathy in cancer patients.

In conclusion, this RT provides a comprehensive collection on the role of epigenetics and epigenomics in cancer drug discovery and development, providing a detailed view of the field, from basic principles to applications in cancer disease therapeutics.
